# Enhanced immune response by vacuoles isolated from *Saccharomyces cerevisiae* in RAW 264.7 macrophages

**DOI:** 10.1042/BSR20211158

**Published:** 2021-09-24

**Authors:** Su-Min Lee, Wooil Choi, Woo-Ri Shin, Yang-Hoon Kim, Jiho Min

**Affiliations:** 1 Graduate School of Semiconductor and Chemical Engineering, Jeonbuk National University, 567 Baekje-daero, Deokjin-Gu Jeonju, Jeonbuk 54896, South Korea; 2 School of Biological Sciences, Chungbuk National University 1 Chungdae-Ro, Seowon-Gu, Cheongju 28644, South Korea

**Keywords:** Cytokine, Immunostimulatory activity, RAW264.7, Saccharomyces cerevisiae, Vacuole

## Abstract

Vacuoles are membrane vesicles in eukaryotic cells, the digestive system of cells that break down substances absorbed outside the cell and digest the useless components of the cell itself. Researches on anticancer and intractable diseases using vacuoles are being actively conducted. The practical application of the present study to animals requires the determination of the biocompatibility of vacuole. In the present study, we evaluated the effects of vacuoles isolated from *Saccharomyces cerevisiae* in RAW 264.7 cells. This showed a significant increase in the production of nitric oxide (NO) produced by macrophage activity. Using Reactive Oxygen Species (ROS) assay, we identified that ROS is increased in a manner dependent on vacuole concentration. Western blot analysis showed that vacuole concentration-dependently increased protein levels of inducible nitric oxide synthase (iNOS), cyclooxygenase-2 (COX-2). Therefore, iNOS expression was stimulated to induce NO production. In addition, pro-inflammatory cytokines levels promoted, such as interleukin (IL) 6 (IL-6) and tumor necrosis factor (TNF) α (TNF-α). In summary, vacuoles activate the immune response of macrophages by promoting the production of immune-mediated transporters NO, ROS, and pro-inflammatory cytokines.

## Introduction

The immune system is a body defense system designed to protect the body from various externally dangerous invaders, such as viruses, bacteria, and microorganisms, such as fungi, bacteria, and parasites [1]. Immune cells can be divided into innate immunity, which is a non-specific natural immunity and adaptive immunity, which is responsible for an immune-specific memory response that is formed by memory in response to pathogens. Innate immune cells first attack foreign substances that have invaded the body, and transfer the information obtained from macrophages to the acquired immune cells. Secondly, the T and B cells that received the information attack the second with information about foreign substances that re-infiltrated.

The activation of macrophages plays an important role in regulating inflammation and tissue repair [2]. Macrophages are important components of the immune system, and in addition to acting as host defenses, activated macrophages produce oxidative mediators of reactive oxygen species (ROS) and nitric oxide (NO), and pro-inflammatory cytokines interleukin (IL) or tumor necrosis factor (TNF) [3]. IL is an immunomodulatory cytokine group that is first expressed by leukocytes, of which IL-6 is secreted from various cells, such as macrophages, lymphocytes, and keratinocytes, and is involved in acute inflammatory responses. TNF is a cytokine that is involved in the mechanism of biodefense through inflammation and has antitumor activity and regulation of differentiation proliferation [4].

The vacuole is the most dynamic organelle in eukaryotic cells and is essential for numerous physiological functions. Vacuoles extracted from Yeast *Saccharomyces cerevisiae* are counterparts of mammalian lysosomes. Inside, they have a hydrolase activity optimal at low pH, that plays an important role in the digestion of extraneous foreign substances in cells [5].

Various studies have been conducted for use as a therapeutic agent, such as an ecofriendly antibacterial agent using vacuole, or an anticancer agent using lysosome [6,7]. To apply this treatment to animals or humans, we tried to determine the effect of vacuoles on the immune system. The present study conducted to measure the immune response of macrophages treated with yeast vacuoles, an environment-friendly substance, against RAW 264.7 macrophages. The effect of activating the immune response of RAW 264.7 cells treated with yeast vacuoles was investigated measuring inflammatory mediators such as NO, ROS and inducible nitric oxide synthase (iNOS), COX 2 protein, and the pro-inflammatory cytokines TNF-α and IL-6.

## Materials and methods

### Materials

Fetal bovine serum (FBS) and Dulbecco’s modified Eagle’s medium (DMEM) were purchased from Gibco (Life Technologies Corp., U.S.A.). 3-(4,5-Dimethylthiazol-2-yl)-2,5-diphenyltetrazolium bromide (MTT), lipopolysaccharide (LPS, *Escherichia coli* O111:B4), and Dexamethasone (DEX) were purchased from Sigma Chemical Co. (St. Louis, MO, U.S.A.). Enzyme-linked immunosorbent assay (ELISA) kits of TNF-α and IL-6 were purchased from R&D Systems (Minneapolis, MN, U.S.A.).

### Isolation of vacuole from *S. cerevisiae*

Yeast *S. cerevisiae* was cultured in YPD medium (1% yeast extract, 2% peptone, and 2% d-glucose) at an *OD_600_* of 0.8–0.9, and the culture was obtained by centrifugation at 3500 rpm for 5 min. And recombinant yeast was grown in synthetic dropout (SD) medium at 30°C incubator. After 24 h of growth in SD medium (0.67% yeast nitrogen base, 0.5% casamino acid, and 2% d-glucose), 2% d-galactose was added and incubated at 180 rpm for 20 h. After adding 0.1 M Tris-SO_4_ buffer (Tris-SO_4_ (pH 9.4), 10 mM dithiothreitol (DTT)) to the obtained cells, the cell walls of the yeast were incapacitated by incubation at 90 rpm for 15 min in a 30°C incubator. The reaction was completed, the cells were centrifuged at 3000 rpm for 5 min, and the supernatant was discarded. The glass beads were added equal to the cell amount, and resuspended in a breaking buffer (20 mM Tris-HCl (pH 7.4), 0.6 M sorbitol, 1 mM phenyl methane sulfonyl fluoride (PMSF)), followed by vortexing ten-times for 1 min (on/off) for a total of 20 min. After centrifugation at 500×***g*** for 10 min, the supernatant was subdivided into a microtube, and then centrifugation was conducted at 20000×***g*** for 30 min by microcentrifuge. After that, the experiment was progressed out using the obtained pellet. For quantification of the concentration of yeast-derived vacuoles, the enzyme contained in the yeast was extracted through the lysis process. After mixing the obtained vacuole and Lysis buffer (0.1% NP-40, 0.1 mM PMSF, 5 mM DTT) in a 1:1 ratio, followed by vortexing ten-times for 10 s (on/off) for a total of 10 min. After that, it was incubated on ice for 30 min. By measuring the protein concentration of the obtained protein through the Bradford assay, the concentration of the vacuole was traced back.

### Observation of the morphologies of vacuole by Field Emission Scanning Electron Microscopy

The vacuole morphology from *S. cerevisiae* was analyzed through Field Emission Scanning Electron Microscopy (FE-SEM) images. The obtained vacuoles were diluted 1:100 in DW and lyophilized for 4 h based on a total volume of 100 µl to form a powder. The prepared sample was observed through FE-SEM (SUPRA40VP, Carl Zeiss, Germany) installed at the Center of University-wide Research Facilities at Jeonbuk National University.

### Cell culture and cell viability assay

The mouse monocyte macrophages cell line RAW 264.7 cells were purchased from the Korean Cell Line Bank (KCLB) and were cultured in DMEM supplemented with 10% FBS, 100 U/ml penicillin, and 100 µg/ml streptomycin in a humidified 5% CO_2_ atmosphere at 37°C. MTT assay was performed to investigate the effect of vacuoles extracted from *S. cerevisiae* on the viability of RAW 264.7 cells [8]. RAW 264.7 cells were seeded in a 24-well plate (1 × 10^5^ cells/well), and cultured for 24 h in DMEM-included 10% FBS. After treatment, different concentrations of vacuole and LPS (1 µg/ml) or DEX (1 µg/ml) were incubated for 24 h, and then 5 mg/ml of MTT solution was added for 2 h. Finally, the supernatant was removed, and formazan crystals were dissolved, using dimethyl sulfoxide (DMSO). Absorbance was measured at 540 nm by an ELISA plate reader. The group treated with LPS (1 µg/ml) was set as a positive control, while the group treated with DEX (1 µg/ml) was set as a negative control.

### Measure of nitrite

The NO level released from the medium was measured using Griess Reagent System (Promega Co., Ltd., U.S.A.). RAW 264.7 cells were cultured in 100-mm dishes at 2 × 10^6^ cells/well and with vacuole and LPS (1 µg/ml) or DEX (1 µg/ml), were incubated for 24 h. The mixture was mixed with a solution of sulfanilamide in equal volume as 50 µl of the culture supernatant, and reacted for 10 min. Afterward, 50 µl of the N-1-naphthylethylenediamine dihydrochlorid (NED) solution was dispensed in each well. Absorbance was measured at 540 nm with an ELISA plate reader. The positive group was set as the LPS (1 µg/ml) treatment group.

### Western blot analysis

RAW 264.7 cells (2 × 10^6^ cells/well) were seeded in 100-mm dishes and incubated for 24 h. After starvation for 12 h in serum-free DMEM, they were treated with each concentration of vacuole and LPS (1 µg/ml), and DEX (1 µg/ml) for 24 h. After removing the medium, cells were washed twice with cold DPBS. They were then lysed with cell lysis buffer, and 20 µg of protein concentration was quantified by Bradford assay. Proteins were separated in SDS/PAGE and transferred from gel to nitrocellulose membranes by electroblotting. Then membranes were blocked in 5% skim milk in T-TBS for 1 h. The iNOS (Abcam, 1:3000) and cyclooxygenase-2 (COX-2) (Abcam, 1:5000) primary antibodies were diluted in 5% skim milk and incubated overnight at 4°C. The membranes were washed with TBS-T and reacted with secondary antibody (Abcam, 1:5000) in 5% skim milk at room temperature (RT) for 1 h. The target protein was confirmed by enhanced chemiluminescence (Dyne Bio, Korea). The positive group was set as the LPS (1 µg/ml) treatment group.

### Secretion of TNF-α, IL-6

After treatment for each concentration of vacuole, levels of TNF- α and IL-6 were measured using an ELISA kit (R&D system, Minneapolis, MN, U.S.A.). After adding 50 µl of ELISA diluent to each well of the antibody-coated 96-well plate, 50 µl of sample and diluted standards were added, and cells were incubated for 2 h at RT, then washed five-times with washing buffer. Enzyme working reagent was added to each well of 100 µl and incubated for 30 min at RT. After washing a total of seven-times, 100 µl of TMB One-Step Substrate Reagent was added, and the plate was incubated for 30 min at RT. After adding 50 µl of stop solution, the absorbance was measured at 450 nm with an ELISA plate reader. The positive group was set as the LPS (1 µg/ml) treatment group.

### Measure of ROS generation

The degree of reactive oxidative species (ROS) occurrence was determined using the 2′,7′-dichlorofluorescin diacetate (DCFH-DA) staining method. RAW 264.7 cells were cultured in six-well plates (1.5 × 10^5^ cells/well) for 24 h. After treatment of vacuole by concentration, the cells were incubated for 24 h. After treatment with 10 µM DCFH-DA, cells were protected from light and incubated for 1 h. After the obtained cells were scraped, the Lysis buffer (20 mM Tris-Cl (pH 7.4), 1 mM EDTA (pH 8.0), 150 mM NaCl, 1 mM EGTA (pH 8.5), 1% (v/v) Triton X-100) was dispensed in 60-µl increments, followed by a vortexing process of 10 s (on/off) for a total of 10 min. Then DCFH-DA fluorescence was measured using GloMax® Explorer Multimode Microplate Reader (emission filter 500–550, excitation filter: blue 475 nm).

### ROS-generated fluorescence imaged using confocal laser scanning microscopy

RAW 264.7 cells were cultured in six-well plates (1.5 × 10^5^ cells/well) on a cover glass for 24 h. After treatment of vacuole by concentration, the cells were incubated for 24 h. Then, 10 µM DCFH-DA was protected from light and incubated for 2 h. After washing once with DPBS, it was treated with Lyso-tracker 100 nm concentration for 20 min. Samples were fixed in 4% paraformaldehyde for 10 min at RT and then analyzed by confocal laser scanning microscopy (LSM 880 with Airyscan, Carl Zeiss, Germany).

### Data analysis

Each data point was obtained from three independent samples conducted simultaneously for error analysis. The averages are reported with the standard deviations and correlations for several experimental conditions. The data were analyzed using a one-way ANOVA and Tukey’s test using SigmaPlot (Systat Software, Inc., U.S.A.). A *P*-value <0.05 was considered significant.

## Results

### Effect of vacuole on cell viability

**Figure 1:**
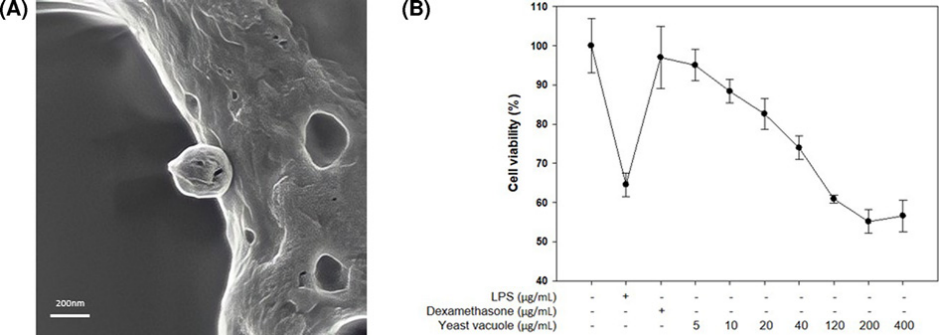
Effect of vacuoles on the cell viability of RAW 264.7 cells (**A**) FE-SEM imagery of vacuole extracted from *S. cerevisiae*. (**B**) Treatment with various concentrations of vacuole for 24 h. LPS (1 µg/ml) was used as a positive control (values are the mean ± SD of three independent experiments).

The vacuoles extracted from yeast *S. cerevisiae* was observed by FE-SEM (Figure 1A). The vacuoles identified by FE-SEM were observed to have a round shape and a size of 200 nm. MTT assay was performed to evaluate the cytotoxicity of vacuole extracted from *S. cerevisiae* to RAW 264.7 macrophages (Figure 1B). Vacuoles were treated at various concentrations of (5, 10, 20, 40, 120, 200, and 400) µg/ml in RAW 264.7 cells, and incubated for 24 h. Figure 1B shows that the vacuoles revealed cell viability of (81.42 ± 5.3)% at 20 µg/ml concentration, and a cell viability of (76.88 ± 2.17)% at 40 µg/ml concentration compared with the control group. Therefore, vacuole with cell viability of 80% or higher were selected for subsequent experiments at a concentration of 20 µg/ml or less.

### Effect of vacuole on the NO production and protein expression of iNOS and COX-2

**Figure 2:**
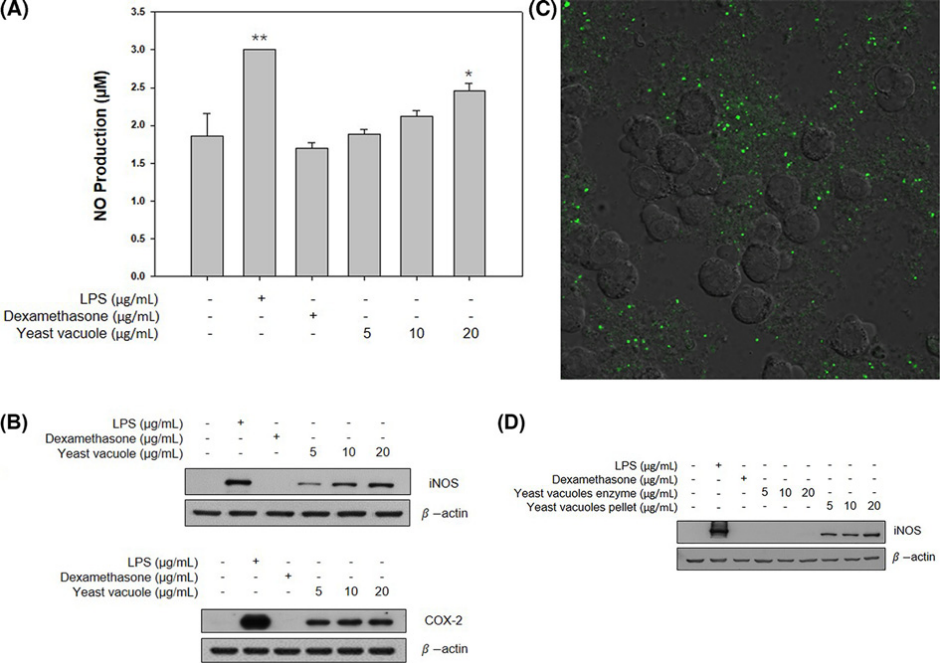
Effect of vacuoles on the production of inflammatory mediator Effect of vacuole on (**A**) NO production, (**B)** iNOS and COX-2 protein levels of RAW 264.7 cells. (**C**) Treatment with GFP-vacuole; confocal scan confirmed that it did not enter RAW 264.7 cells. (**D**) Effect of vacuole enzymes and pellets on iNOS protein level of RAW 264.7 cells. * represents *P*-value <0.05, ** represents *P*-value <0.002 compared with the control group. LPS (1 µg/ml) was used as a positive control (values are the mean ± SD of the three independent experiments).

NO is a cell-signaling molecule that works in many biological processes [9]. NO, produced by macrophages, is a signal transduction molecule that acts as a defense against infected microorganisms in cells and has been demonstrated to inhibit the proliferation inhibitory activity of bacteria and cancer cells, and non-specific sedative defense mechanisms. Griess assay was performed to investigate the effect of vacuole on NO production in RAW264.7 cells (Figure 2A). Reaction for 24 h after treatment with each concentration (5, 10, and 20 µg/ml) of vacuole and LPS (1 µg/ml) and DEX (1 µg/ml) confirmed that the production of NO increased, depending on the concentration of vacuoles. In addition, NO synthase (NOS) is an enzyme that promotes the production of NO from l-arginine, and can be broadly classified into constitutive NOS (cNOS), and inducible NOS (iNOS). Of these, iNOS is synthesized by inflammatory stimuli and produces large amounts of NO [10]. Then, a Western blot was performed to confirm the effect of vacuole on RAW264.7 macrophages and iNOS and COX-2 protein expression (Figure 2B). The iNOS and COX-2 protein expression levels increased with the concentration of vacuoles, which showed a correlation with NO and iNOS protein expression levels. Furthermore, in order to find out which components of vacuoles extracted from wildtype yeast activate RAW 264.7 cells, enzymes and pellets extracted from vacuoles were treated under equal conditions (Figure 2D). In the group treated with enzyme, the expression of iNOS protein was not observed; but in the group treated with pellet, iNOS protein was expressed. We investigated whether vacuoles penetrate into RAW 264.7 cells by treatment with the vacuole extract of recombinant yeast strain YPT7 (Figure 2C). YPT7 protein is located in the membrane of the vacuole and a transformed yeast recombinant plasmid with GFP attached, pYES2.0::YPT7::GFP was used [7]. At this time, the location of the vacuole can be confirmed through the signal of GFP. When the vacuoles extracted from recombinant yeast were treated in RAW264.7 cells for 24 h, it was confirmed that the vacuoles did not penetrate into the cells. Therefore, the vacuoles were activated by the membrane component of the vacuoles, without penetrating into RAW 264.7 cells.

### Effect of vacuole on the levels of TNF-α and IL-6

**Figure 3:**
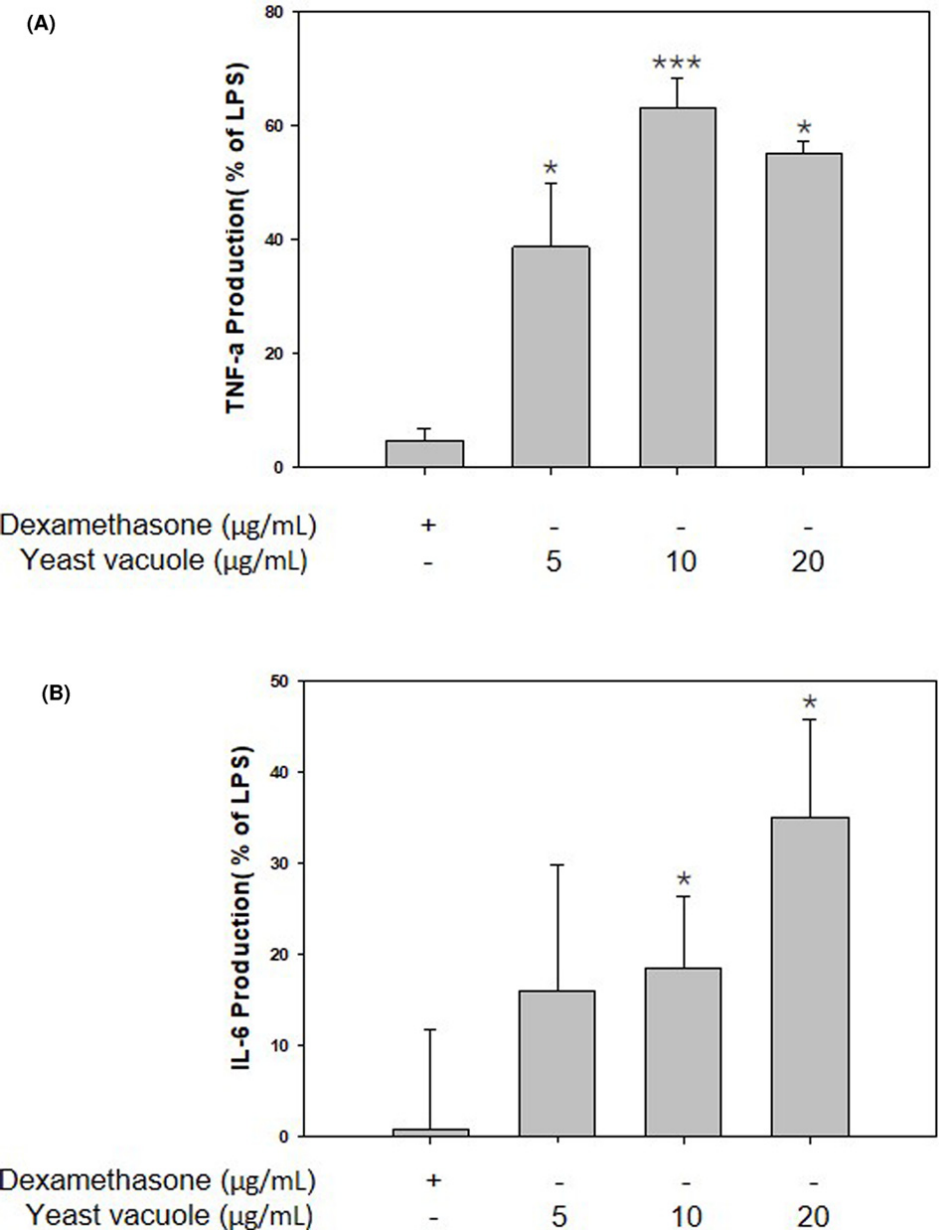
Effect of vacuoles on the production of pro-inflammatory cytokine (**A**) TNF-α, and (**B**) IL-6 secretion by vacuole of RAW 264.7 cells. * represents *P*-value <0.05, *** represents *P*-value <0.005 compared with the control group. LPS (1 µg/ml) was used as a positive control (values are the mean ± SD of three independent experiments).

Cytokines are carriers of the immune system, and are regulatory proteins that can be produced in eukaryotic cell types. They are also a factor that modulate the immune response by acting on the cells and hematopoietic cells involved in the host defense and damage healing process [11]. TNF-α is a cytokine that is mainly produced in activated macrophages, and is responsible for antitumor and antimicrobial activity regulation of differentiation and proliferation [4]. RAW 264.7 cells were treated for 24 h with either vacuoles (5, 10, and 20 μg/ml) or LPS (1 μg/ml) and pro-inflammatory cytokines TNF-α and IL-6 were quantified using a mouse ELISA kit (Figure 3). As a result, vacuoles significantly increased the production of the pro-inflammatory cytokine TNF-α (Figure 3A). It was confirmed that the production of TNF-α by vacuole increased six-fold at a concentration of 10 µg/ml compared with the control group (0 µg/ml vacuole treatment). IL-6 affects adaptive immunity, and is a cytokine related to the proliferation of B cells and the secretion of antibodies, and functions in the immune response and acute inflammatory response [12]. The production of IL-6 by vacuole increased in a concentration-dependent manner (Figure 3B) [13].

### Effect of vacuole on ROS generation in RAW 264.7 cells

**Figure 4:**
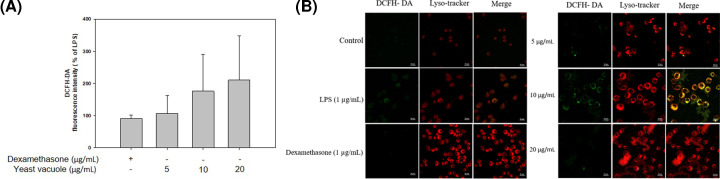
ROS production by vacuoles in RAW 264.7 cells (**A**) RAW 264.7 cells were exposed at vacuoles for 24 h and ROS was detected with DCFH-DA. (**B**) Confocal laser scanning microscopy imaging of the expression level of ROS. LPS (1 µg/ml) was used as a positive control (values at the mean ± SD of three independent experiments).

ROS are produced in processes that are activated in normal cells and are involved in biological processes, including cell differentiation and the degree of response to cytokines [14]. The ROS by vacuole in RAW 264.7 cells was detected using DCFH-DA staining (Figure 4). LPS (1 µg/ml) was set as a positive control. The vacuole treatment group significantly induced the generation of ROS (Figure 4A). As a result of confocal laser scanning microscopy imaging of the expression level of ROS, vacuole concentration was the highest at 10 µg/ml (Figure 4B).

## Discussion

By using the *S. cerevisiae*-derived vacuole used in the present study, it was possible to target cancer and effectively deliver anticancer drugs, and research is being actively conducted as new therapeutic material such as increasing anticancer and antibacterial effects through gene recombination [7,15]. However, studies on the immunological effects of *S. cerevisiae*-derived vacuoles are insufficient. In the present study, the effect of *S. cerevisiae*-derived vacuoles on the immune activation effect of mouse macrophages was investigated. Macrophages are produced from monocytes and are the main cells responsible for innate immunity [16]. Macrophages play several important roles in the immune system. First, free radicals are produced and secreted to kill bacteria, and external bacterial proteins degraded in macrophages are presented on their surface, and information of foreign substances invading T cells is transmitted [17]. Among the immune response mediators produced at this time, various substances such as NO, ROS, histamine, and cytokines are included. The NO produced at this time plays an important role in protecting the body as a cell-signaling molecule. The most important function is to relax the internal muscles of blood vessels by dilating blood vessels, regulate blood pressure, and perform various physiological functions such as inhibition of platelet aggregation, immune regulation, and induction of apoptosis. Although excessively produced NO shows cytotoxicity to normal cells, an appropriate amount of NO has resistance to foreign antigens and plays an important role in host protection [16]. In addition, iNOS is produced in large amounts for host protection when stimulated from the outside, and as a result, the production of NO is induced. In the present study, it was confirmed that when RAW 264.7 cells were treated with vacuoles extracted from *S. cerevisiae*, NO production was promoted and iNOS protein expression also increased in a vacuole concentration-dependent manner (Figure 2A,B). Moreover, among pro-inflammatory cytokines, TNF-α is a cytokine that plays an important role in several immune-mediated inflammatory diseases. TNF-α is mainly produced in activated macrophages and functions to enhance antitumor activity and the ability to kill infected cells [17]. IL-6 is a cytokine that plays an important role in acute-phase response, inflammation, hematopoiesis, and progression of cancer, and performs the body’s defense function against antigens introduced from the outside [18]. In this study, the effect of vacuole on the production of pro-inflammatory cytokines TNF-α and IL-6 was studied. As a result, it was shown that vacuole induces TNF-α and IL-6 production. These results suggest that vacuoles activate macrophages and up-regulate immune capacity. While ROS play an important role in protecting the living body by sterilizing foreign substances invading the body, excessively generated ROS can cause aging and attack normal cells. The vacuoles treatment group significantly induced the generation of ROS. As a result of confirming the generation of ROS by vacuoles through confocal fluorescence images, compared with the LPS-treated group, the expression of ROS was the highest at the concentration of 10 μg/ml of the vacuoles. In the present study, it was confirmed that this immune-activation effect is caused by the membrane component of the vacuole, but it cannot be determined exactly which component is induced in the present study. Activation of macrophages by vacuole membrane components requires additional component analysis studies. Lipopolysaccharide (LPS) used as a positive control in the present study is well used as a strong immune response inducer, but it is difficult to practically apply to the human body as an immune enhancer due to its strong toxicity. Compared with LPS, vacuoles have significantly lower toxicity and are judged to have excellent immune-enhancing effects, suggesting potential as a functional material for strengthening immunity.

## Conclusion

In the present study, after treating vacuoles extracted from *S. cerevisiae* with RAW 264.7 macrophages, cell viability, NO and ROS production, and pro-inflammatory cytokines, such as IL-6 and TNF-α were measured, and the following results were obtained. The cell viability at vacuole concentrations of (5, 10, and 20) µg/ml was greater than 80%. It was confirmed that in the production of NO, the vacuole increased at all concentrations. Vacuole was also correlated with the results of NO in iNOS and COX-2 protein expression. The significant increase in production of pro-inflammatory cytokines IL-6 and TNF-α and ROS at all concentrations of vacuoles was confirmed. Therefore, the vacuole extracted from *S. cerevisiae* suggests that the immune response is activated by promoting the increase in the immune mediator in RAW 264.7 cells.

## Data Availability

All data generated or analyzed during the present study are included in this article.

## Abbreviations

COX-2: cyclooxygenase-2
DCFH-DA: 2′,7′-dichlorofluorescin diacetate
DEX: Dexamethasone
DMEM: Dulbecco’s modified Eagle’s medium
DPBS: Dulbecco's phosphate buffered saline
DTT: dithiothreitol
ELISA: enzyme-linked immunosorbent assay
FBS: fetal bovine serum
FE-SEM: Field Emission Scanning Electron Microscopy
IL: interleukin
iNOS: inducible nitric oxide synthase
MTT: 3-(4,5-dimethylthiazol-2-yl)-2,5-diphenyltetrazolium bromide
NO: nitric oxide
NOS: NO synthase
PMSF: phenyl methane sulfonyl fluoride
ROS: reactive oxygen species
RT: room temperature
SD: synthetic dropout
TNF: tumor necrosis factor
